# Supplementary material for: A simple method for joint evaluation of skill in directional forecasts of multiple variables
Source: Heliyon. 2023 Sep 1;9(9):e19729. doi: 10.1016/j.heliyon.2023.e19729 (PMC10558991; doi:10.1016/j.heliyon.2023.e19729)
Supplement: Multimedia component 1 [file mmc1.pdf]

Supplementary Materials for

A simple method for joint evaluation of skill in directional forecasts of multiple variables

Thitithep Sitthiyot[a,*] and Kanyarat Holasut[b]

[a] Department of Banking and Finance, Faculty of Commerce and Accountancy, Chulalongkorn University, Mahitaladhibesra Bld., 10th Fl., Phayathai Rd., Pathumwan, Bangkok 10330, Thailand.

[b] Department of Chemical Engineering, Faculty of Engineering, Khon Kaen University, Mittapap Rd., Muang District, Khon Kaen 40002, Thailand.

[*] Correspondence to: thitithep@cbs.chula.ac.th.

Table S1. Data on GDP growth forecast of the Bank of Thailand and the actual GDP growth of Thailand between 2001 and 2021. The actual GDP growth in 2000 is included in order to compare the direction-of-change of the actual GDP growth with the forecasts made by the Bank of Thailand in 2001.

**Year**	**GDP growth forecast for the entire year made at the beginning of each year (%)**	**Δ (%)**	**Actual GDP growth (% year-on-year)**	**Δ (%)**
2000	-	-	4.80	-
2001	3.75	-	2.20	-2.60
2002	2.50	-1.25	5.30	3.10
2003	4.00	1.50	7.10	1.80
2004	6.80	2.80	6.30	-0.80
2005	5.80	-1.00	4.60	-1.70
2006	5.25	-0.55	5.10	0.50
2007	4.50	-0.75	5.00	-0.10
2008	5.25	0.75	2.50	-2.50
2009	1.00	-4.25	-2.30	-4.80
2010	4.30	3.30	7.80	10.10
2011	4.00	-0.30	0.10	-7.70
2012	4.90	0.90	6.50	6.40
2013	4.90	0.00	2.90	-3.60
2014	2.70	-2.20	0.80	-2.10
2015	3.80	1.10	2.90	2.10
2016	3.10	-0.70	3.30	0.40
2017	3.40	0.30	4.00	0.70
2018	4.10	0.70	4.20	0.20
2019	3.80	-0.30	2.40	-1.80
2020	2.80	-1.00	-6.10	-8.50
2021	3.20	0.40	1.60	7.70

Sources: 1. Bank of Thailand, 2022. Monetary Policy Report. https://www.bot.or.th/English/MonetaryPolicy/MonetPolicyComittee/MPR/Pages/default.aspx.

2. Office of the National Economic and Social Development Council, 2022. Thai Economic Performance. https://www.nesdc.go.th/main.php?filename=macroeconomics.

Table S2. Data on GDP growth forecast of the Fiscal Policy Office and the actual GDP growth of Thailand between 2003 and 2021. The actual GDP growth in 2002 is included in order to compare the direction-of-change of the actual GDP growth with the forecasts made by the Fiscal Policy Office in 2003.

Year	GDP growth forecast for the entire year made at the beginning of each year (%)	Δ (%)	Actual GDP growth (% year-on-year)	Δ (%)
2002	-	-	5.30	-
2003	5.10	-	7.10	1.80
2004	7.90	2.80	6.30	-0.80
2005	6.00	-1.90	4.60	-1.70
2006	5.00	-1.00	5.10	0.50
2007	4.25	-0.75	5.00	-0.10
2008	5.50	1.25	2.50	-2.50
2009	-2.50	-8.00	-2.30	-4.80
2010	4.50	7.00	7.80	10.10
2011	4.50	0.00	0.10	-7.70
2012	5.00	0.50	6.50	6.40
2013	5.00	0.00	2.90	-3.60
2014	4.00	-1.00	0.80	-2.10
2015	3.90	-0.10	2.90	2.10
2016	3.70	-0.20	3.30	0.40
2017	3.60	-0.10	4.00	0.70
2018	4.20	0.60	4.20	0.20
2019	4.00	-0.20	2.40	-1.80
2020	2.80	-1.20	-6.10	-8.50
2021	2.80	0.00	1.60	7.70

Sources: 1. Fiscal Policy Office, 2022. Thailand Economic Outlook. http://www.fpo.go.th/main/Economic-report/Thailand-Economic-Projections.aspx.

2. Office of the National Economic and Social Development Council, 2022. Thai Economic Performance. https://www.nesdc.go.th/main.php?filename=macroeconomics.

Table S3. Data on GDP growth forecast of Office of the National Economic and Social Development Council (NESDC) and the actual GDP growth of Thailand between 2001 and 2021. The actual GDP growth in 2000 is included in order to compare the direction-of-change of the actual GDP growth with the forecasts made by the NESDC in 2001.

Year	GDP growth forecast for the entire year made at the beginning of each year (%)	Δ (%)	Actual GDP growth (% year-on-year)	Δ (%)
2000	-		4.80	
2001	4.00		2.20	-2.60
2002	2.00	-2.00	5.30	3.10
2003	4.00	2.00	7.10	1.80
2004	7.50	3.50	6.30	-0.80
2005	6.00	-1.50	4.60	-1.70
2006	4.70	-1.30	5.10	0.50
2007	4.50	-0.20	5.00	-0.10
2008	4.50	0.00	2.50	-2.50
2009	3.50	-1.00	-2.30	-4.80
2010	3.50	0.00	7.80	10.10
2011	4.00	0.50	0.10	-7.70
2012	5.00	1.00	6.50	6.40
2013	3.00	-2.00	2.90	-3.60
2014	3.50	0.50	0.80	-2.10
2015	4.00	0.50	2.90	2.10
2016	3.30	-0.70	3.30	0.40
2017	3.50	0.20	4.00	0.70
2018	4.10	0.60	4.20	0.20
2019	4.00	-0.10	2.40	-1.80
2020	3.20	-0.80	-6.10	-8.50
2021	3.00	-0.20	1.60	7.70

Source: Office of the National Economic and Social Development Council, 2022. Thai Economic Performance. https://www.nesdc.go.th/main.php?filename=macroeconomics.

Table S4. Data on inflation forecast of the Bank of Thailand and the actual inflation of Thailand between 2001 and 2021. The actual inflation in 2000 is included in order to compare the direction-of-change of the actual inflation with the forecasts made by the Bank of Thailand in 2001.

**Year**	**Inflation forecast for the entire year made at the beginning of each year (%)**	**Δ (%)**	**Actual inflation (% year-on-year)**	**Δ (%)**
2000	-	-	1.60	-
2001	2.00	-	1.60	0.00
2002	0.50	-1.50	0.70	-0.90
2003	1.00	0.50	1.80	1.10
2004	1.50	0.50	2.70	0.90
2005	3.00	1.50	4.50	1.80
2006	4.25	1.25	4.70	0.20
2007	1.75	-2.50	2.30	-2.40
2008	3.40	1.65	5.50	3.20
2009	-0.50	-3.90	-0.90	-6.40
2010	4.00	4.50	3.30	4.20
2011	3.50	-0.50	3.80	0.50
2012	3.20	-0.30	3.00	-0.80
2013	2.80	-0.40	2.20	-0.80
2014	2.50	-0.30	1.90	-0.30
2015	0.20	-2.30	-0.90	-2.80
2016	0.60	0.40	0.19	1.09
2017	1.20	0.60	0.66	0.47
2018	1.00	-0.20	1.07	0.41
2019	1.00	0.00	0.71	-0.36
2020	0.80	-0.20	-0.80	-1.51
2021	1.00	0.20	1.20	2.00

Source: Bank of Thailand, 2022. Monetary Policy Report. https://www.bot.or.th/English/MonetaryPolicy/MonetPolicyComittee/MPR/Pages/default.aspx.

Table S5. Data on inflation forecast of the Fiscal Policy Office and the actual inflation of Thailand between 2003 and 2021. The actual inflation in 2002 is included in order to compare the direction-of-change of the actual inflation with the forecasts made by the Fiscal Policy Office in 2003.

Year	Inflation forecast for the entire year made at the beginning of each year (%)	Δ (%)	Actual inflation (% year-on-year)	Δ (%)
2002	-	-	0.70	-
2003	2.30	-	1.80	1.10
2004	2.40	0.10	2.70	0.90
2005	4.10	1.70	4.50	1.80
2006	3.00	-1.10	4.70	0.20
2007	2.80	-0.20	2.30	-2.40
2008	4.50	1.70	5.50	3.20
2009	0.70	-3.80	-0.90	-6.40
2010	4.00	3.30	3.30	4.20
2011	3.60	-0.40	3.80	0.50
2012	3.50	-0.10	3.00	-0.80
2013	3.00	-0.50	2.20	-0.80
2014	2.40	-0.60	1.90	-0.30
2015	0.90	-1.50	-0.90	-2.80
2016	0.30	-0.60	0.19	1.09
2017	1.80	1.50	0.66	0.47
2018	1.20	-0.60	1.07	0.41
2019	1.00	-0.20	0.71	-0.36
2020	0.80	-0.20	-0.80	-1.51
2021	1.30	0.50	1.20	2.00

Sources: 1. Fiscal Policy Office, 2022. Thailand Economic Outlook. http://www.fpo.go.th/main/Economic-report/Thailand-Economic-Projections.aspx.

2. Bank of Thailand., 2022. Monetary Policy Report. https://www.bot.or.th/English/MonetaryPolicy/MonetPolicyComittee/MPR/Pages/default.aspx.

Table S6. Data on inflation forecast of Office of the National Economic and Social Development Council (NESDC) and the actual inflation of Thailand between 2001 and 2021. The actual inflation in 2000 is included in order to compare the direction-of-change of the actual inflation with the forecasts made by the NESDC in 2001.

Year	Inflation forecast for the entire year made at the beginning of each year (%)	Δ (%)	Actual inflation (% year-on-year)	Δ (%)
2000	-	-	1.60	-
2001	2.25	-	1.60	0.00
2002	2.00	-0.25	0.70	-0.90
2003	1.50	-0.50	1.80	1.10
2004	2.50	1.00	2.70	0.90
2005	3.05	0.55	4.50	1.80
2006	4.00	0.95	4.70	0.20
2007	3.40	-0.60	2.30	-2.40
2008	3.25	-0.15	5.50	3.20
2009	3.00	-0.25	-0.90	-6.40
2010	3.00	0.00	3.30	4.20
2011	3.00	0.00	3.80	0.50
2012	3.75	0.75	3.00	-0.80
2013	3.00	-0.75	2.20	-0.80
2014	2.40	-0.60	1.90	-0.30
2015	0.50	-1.90	-0.90	-2.80
2016	0.40	-0.10	0.19	1.09
2017	1.70	1.30	0.66	0.47
2018	1.40	-0.30	1.07	0.41
2019	1.00	-0.40	0.71	-0.36
2020	0.90	-0.10	-0.80	-1.51
2021	1.50	0.60	1.20	2.00

Sources: 1. Office of the National Economic and Social Development Council, 2022. Thai Economic Performance. https://www.nesdc.go.th/main.php?filename=macroeconomics.
2. Bank of Thailand, 2022. Monetary Policy Report. https://www.bot.or.th/English/MonetaryPolicy/MonetPolicyComittee/MPR/Pages/default.aspx.
